# Mechanistic causes of sign epistasis and its applications

**DOI:** 10.3389/fgene.2024.1366917

**Published:** 2024-02-28

**Authors:** Jinqiu Zhang, Feiyu Chen, Xianghua Li

**Affiliations:** ^1^ Zhejiang University—University of Edinburgh Institute, Zhejiang University, Haining, China; ^2^ Faculty of Biomedical Sciences, School of Medicine and Veterinary Medicine, University of Edinburgh Institute, Edinburgh, United Kingdom; ^3^ Wellcome Sanger Institute, Wellcome Genome Campus, Hinxton, United Kingdom

**Keywords:** epistasis, sign epistasis, genetics, genetic mutations, genotype-phenotype mapping

## Abstract

Mapping genetic variations to phenotypic variations poses a significant challenge, as mutations often combine unexpectedly, diverging from assumed additive effects even in the same environment. These interactions are known as epistasis or genetic interactions. Sign epistasis, as a specific type of epistasis, involves a complete reversal of mutation effects within altered genetic backgrounds, presenting a substantial hurdle to phenotype prediction. Despite its importance, there is a limited systematic overview of the mechanistic causes of sign epistasis. This review explores the mechanistic causes, highlighting its occurrence in signalling cascades, peaked fitness landscapes, and physical interactions. Moving beyond theoretical discussions, we delve into the practical applications of sign epistasis in agriculture, evolution, and antibiotic resistance. In conclusion, this review aims to enhance the comprehension of sign epistasis and molecular dynamics, anticipating future endeavours in systematic biology engineering that leverage the knowledge of sign epistasis.

## Introduction

The mapping of genotype-to-phenotype has been at the core of genetics, yet the relationship between them remains complex and challenging to predict. Even in controlled environments, mutations often interact unexpectedly, deviating from the conventional assumption of additive mutational effects, a phenomenon referred to as genetic interactions or epistasis (Fisher, 1919; Phillips, 2008; Domingo et al., 2019).

Among various types of epistasis, sign epistasis, as a severe form, poses the greatest challenge to phenotype prediction and thus warrants special attention. Sign epistasis occurs when the effect of one mutation completely switches direction from positive to negative, and *vice versa*, within altered genetic backgrounds (Weinreich et al., 2005). This phenomenon can manifest within a single gene or between different genes. While not always anticipated, such occurrences are common (de Visser et al., 2011; Poelwijk et al., 2011) and significantly constrain evolutionary paths (Weinreich et al., 2005; Weinreich et al., 2006). For example, sign epistasis plays a crucial role in protein evolution, where negative sign epistasis may lead to evolutionary dead ends, and negative reciprocal sign epistasis is responsible for the divergence and branching of evolutionary pathways (Miton et al., 2021; Buda et al., 2023).

**FIGURE 1:**
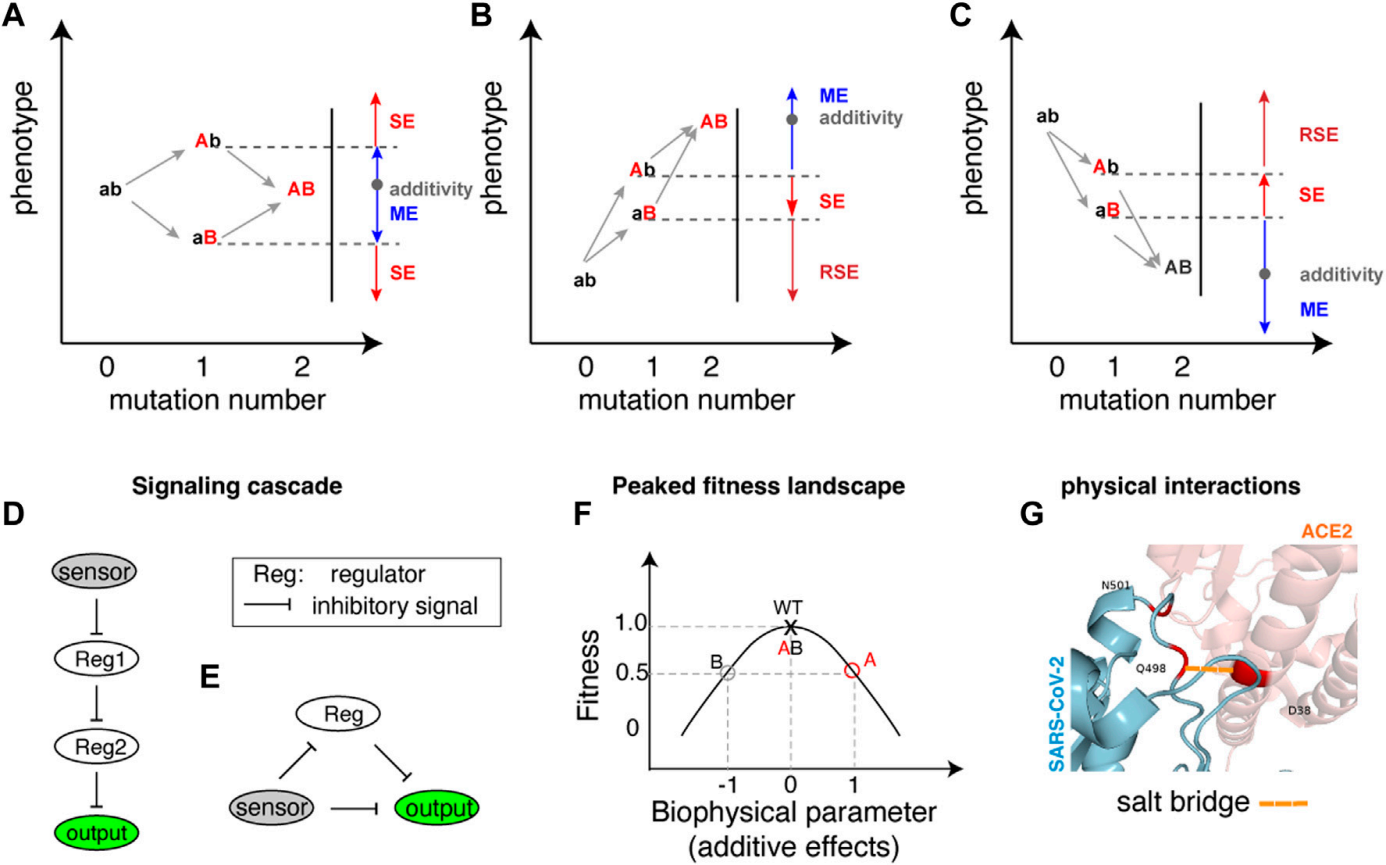
Illustration of sign epistasis and mechanisms. **(A–C)** Sign epistasis in the perspective of double mutations in different mutation combination scenarios where one mutation is detrimental (lower phenotype) while the other mutation is beneficial (higher phenotype) **(A)**, both single mutations are beneficial **(B)** and both single mutations are detrimental **(C)**. The blue colour represents Magnitude Epistasis (ME), red indicates Sign Epistasis (SE), and dark red signifies Reciprocal Sign Epistasis (RSE). Additivity (shown as a grey dot in each panel) implies that the phenotype resulting from a double mutation equals the sum of phenotypic changes by individual mutations. **(D, E)** Architectures of gene regulatory networks generating sign epistasis, with D and E showcasing different architectures in *E. coli*. **(F)** A peaked fitness landscape can generate sign epistasis. The red and grey circles each represent single mutations A and B with fitness values of 0.5 compared to the wildtype fitness value of 1. The horizontal arrows indicate biophysical parameter changes in two opposite directions. The double mutant AB fitness becomes the same as the wild type due to the combined effect of underlying biochemical parameters. **(G)** An example of protein residue physical interactions at the binding interface generating sign epistasis. A salt bridge is highlighted in a dashed line coloured in orange shade.

Regardless of whether single mutations have detrimental or beneficial effects individually, their combinations can result in sign epistasis. Here, we categorise the occurrence of sign epistasis into three types based on the effects of the single mutations, as depicted in Figure 1.

For scenarios where a beneficial (Mutation A) and a detrimental (Mutation B) mutation combine (Figure 1A), sign epistasis occurs if the combined phenotype (AB) surpasses the beneficial effect of the single mutant (Ab). In this case, Mutation B changes its sign from negative to positive in the presence of Mutation A. Conversely, if the double mutant (AB) effect is inferior to the detrimental effect of the single mutant (aB), Mutation A changes its sign while Mutation B retains its negative effect. The other cases of deviation from additivity, termed magnitude epistasis (ME), occur when the double mutants’ phenotype falls within the boundary defined by the two single mutations (Figure 1A).

When two beneficial mutations combine (Figure 1B), sign epistasis arises if the combined effects are worse than the better phenotype of the two single mutations. As illustrated in Figure 1B, if the double mutant (AB) phenotype falls between the two single mutants, the less beneficial mutant (Mutation B) changes its sign from beneficial to detrimental in the presence of the more beneficial mutant (Mutation A). If the double mutant (AB) phenotype is not as good as the less beneficial mutant (Mutation B), it indicates reciprocal sign epistasis (RSE), where both Mutation A and B independently exhibit sign epistasis effects, showcasing opposite effects when the other mutation is present.

Combining two detrimental mutations (Figure 1C) can also lead to both sign and reciprocal sign epistasis, with the combined effect surpassing either one of the single mutational effects.

The exploration of sign epistasis in quantitative genetics unravels a complex tapestry of genetic interactions, molecular dynamics, and broader implications across various biological systems. Despite the unique characteristics of sign epistasis compared to other forms of epistasis, its causes have seldom been systematically discussed separately from general epistasis (Phillips, 2008; de Visser et al., 2011; Domingo et al., 2019).

In this review, we elucidate the mechanistic causes of sign epistasis and discuss its broader implications in the realm of genetics.

## Signaling cascade generates sign epistasis between genes

Epistasis between genes is often generated from the upstream-downstream relationship between genes, and recent studies highlight that these relationships also produce sign epistasis.

The occurrence of sign epistasis was demonstrated in a synthetic signalling cascade in bacteria (Figure 1D), consisting of a sensor for induction signal (arabinose for instance), repressors (tetR and lacI as two regulators for instance) and a reporter gene (YFP), (Nghe et al., 2018). Within this gene regulatory network, the two repressors (shown as Reg1 and Reg2 in Figure 1D) coordinate the integration of the induction signal to gene expression hierarchically. Mutations introduced to either upstream or downstream regulatory repressors, or both could switch the direction of mutational effects, resulting in sign epistasis. Biochemical modelling reveals that specific combinations of mutations on transcription factors’ binding affinities to response elements predispose the system to exhibit sign epistasis (Nghe et al., 2018). In essence, sign epistasis is anticipated when certain mutation combinations are introduced to both the upstream and downstream genes in a hierarchical signalling cascade.

Another architecture of synthetic network using the same components (sensing arabinose, regulation via tetR and lacI, controlling reporter gene) in the bacterial system can also lead to the frequent manifestation of sign epistasis(Figure 1E). Unlike the linear upstream-downstream cascade, this synthetic gene regulatory network involves direct inhibitory control and delayed positive regulation (via double inhibitory regulation) of an output (Figure 1E). In this system that leads to a peaked response with increasing inducer concentration, over 50% of the significant epistatic pairs exhibit sign epistasis when two mutations are introduced into two of the three components. Notably, when two mutations enhancing output gene expression combine (as depicted in Figure 1B), the majority of significant epistatic pairs manifest as negative reciprocal sign epistasis pairs (Baier et al., 2023). This observation suggests that combinations of beneficial mutations in two genes can frequently result in detrimental effects in such gene regulatory networks.

## Peaked fitness landscapes generate sign epistasis between and within genes

Many biological systems exhibit peaked fitness landscapes that are not monotonically related to gene expression levels or protein activities (Keren et al., 2016), or inducer signals (as the gene regulatory network depicted in Figure 1E). Due to the non-monotonic nature of the system (i.e., a non-monotonic relationship between the protein activity and fitness), combining the two mutations can often produce unexpected results—sign epistasis. Such phenomena have not only been theorized (Gjuvsland et al., 2013) but also observed frequently. Metabolic flux systems, featuring multiple enzymes, commonly generate peaked fitness landscapes (Dykhuizen et al., 1987). Examples include frequent sign epistasis observed between flhA and fghA genes within the GSH-linked metabolic pathway (Chou et al., 2014) and between araA and araB genes in the Arabinose utilization pathway (Chou et al., 2014; Kemble et al., 2020).

Figure 1F illustrates how a peaked fitness landscape generates sign epistasis. As shown in Figure 1F, the wildtype sits on the peak of the fitness landscape, and two detrimental mutations A and B each decrease the fitness to half of the wildtype value (0.5 in the y-axis). Although the fitness effects of the two mutations are the same, their effects on underlying biophysical parameters or protein activity/levels can be opposite (shown in the x-axis), the combined effect pushes the protein activity back to the fitness peak, resulting in seemingly sign epistatic effect (Figure 1F). Such examples can be found in maintaining balanced levels of autophagy, where both insufficient and excessive amounts can lead to detrimental effects (Kang and Avery, 2008).

Even in the context of a single gene system, the expression-fitness landscapes (Keren et al., 2016) and protein stability-fitness relationship (DePristo et al., 2005) can take on a peaked form. Studies on protein folding energy unveil a neutral range, typically within 1 kcal/mol^-1^ of the wildtype stability (DePristo et al., 2005). Decreased stability beyond this range may lead to reduced protein concentration, while increased stability beyond the range could result in aggregation and, thus, reduced fitness. Consequently, a mutation could have either a positive or negative impact depending on the stability of the genetic background of the protein (DePristo et al., 2005). Although we did not find experimentally demonstrated examples, it is theoretically plausible to expect that mutations within the same gene, each altering expression level, could generate sign epistasis (Li and Lehner, 2020).

## Physical interactions generate sign epistasis between and within genes

Physical interactions among atoms within a protein or a protein complex lead to sign epistasis (Figure 1G). Sign epistasis induced by structural contacts can be considered a subcategory of specific epistasis (Domingo et al., 2019), sometimes referred to as contact epistasis (Wonderlick et al., 2023) or idiosyncratic epistasis (Johnson et al., 2023), signifying epistasis resulting from structural interactions between two residues within the protein’s configuration.

Taking the SARS-CoV-2 mutation Q498R as an example, this mutation weakly reduces the binding affinity of the alpha variant to its receptor (ACE2) but enhances the binding affinity with the N501Y mutation (Starr et al., 2022). Molecular dynamic simulations have revealed that the Q498R and N501Y mutations collectively restore individually disrupted salt bridges. Additionally, they establish a new salt bridge with the D38 residue of the ACE2 receptor, augmenting its receptor-bound stability (Starr et al., 2022). The coexistence of both mutations induces a significant alteration in the protein’s structural configuration. A parallel scenario is observed with the HIV-1 protease gene, where a detrimental mutation (L10I) enhances fitness when G48V and L90M mutations are present through structural modification of the encoded protein (Mammano et al., 2000).

Sign epistasis of this kind also frequently occurs between two mutations, each in one of the two interacting molecules. A classic example is evident in the barnase and barstar protein-protein interaction model system, where two individually detrimental mutations (E76R in barstar and R59E in barnase) combine to restore the stable complex through interchanging charges between the interacting positions (Jucovic and Hartley, 1996). Bacterial toxin-antitoxin pairs also exhibit abundant sign epistasis (Aakre et al., 2015) via the same mechanism.

Besides frequently observed in protein coding genes, this type of sign epistasis arising from structural contacts is also observed for RNA phenotypes, such as mutational effects on alternative splicing (Baeza-Centurion et al., 2019) and tRNA function (Domingo et al., 2018).

## Application of sign epistasis

In the realm of agriculture, artificial intervention, including artificial selection for favourable traits, is crucial. However, the genetic architecture and the causal effects of genetic interaction on traits of interest are often overlooked, leading to unexpected variance in crop yields (Goldringer et al., 1997; Blanc et al., 2006) and maize flowering (Buckler et al., 2009). The significance of sign epistasis, with its sign-reverting property on different genetic backgrounds, lies in trait selection and optimization. Studies by Vagne and colleagues (Vagne et al., 2015) emphasize the importance of introducing reciprocal sign epistasis into analysis of critical recombination rates to fix optimal genotypes. This shed light on a strategy to better understand the relationship between key parameters and the fixation of fittest traits. Sign epistasis can also contribute to maximizing heterosis, indicating its potential utility for optimizing traits in crops.

Sign epistasis between alleles can even influence the formation of nascent species. Postzygotic reproductive isolation, a key factor in this context, is often attributed to the Bateson-Dobzhansky-Muller (BDM) incompatibilities (Orr, 1995; Orr, 1996; Presgraves, 2010). The genetics underlying these incompatibilities align with reciprocal sign epistasis, where the introduction of alleles from other species underperforms alleles with favoured phenotypes in hybrid individuals, resulting in hybrid infertility or lethality (Ono et al., 2017).

Furthermore, the nature of sign epistasis plays a crucial role in shaping the broad-scale evolutionary phenomenon in terms of the shape and “ruggedness” of the fitness landscape (Poelwijk et al., 2007; de Visser et al., 2011; Poelwijk et al., 2011). Hypothetically, if all possible alleles exert the same effects across all genetic backgrounds, the population’s variance is expected to converge toward a similar genotype of optimal fitness. Conversely, when some alleles are only beneficial or harmful in specific genetic backgrounds, the chronological order of genetic changes may result in a diverse population both genetically and phenotypically. Viral genomes appear to be dominated by sign epistasis (Molla et al., 1996; Cong et al., 2007; Martinez-Picado and Martínez, 2008; Lalić and Elena, 2012), indicating the significant role sign epistasis plays in viral genome evolution.

Sign epistasis is also prevalent in bacteria, offsetting the cost of antibiotic resistance including resistance to nalidixic acid (gyrA mutation), rifampicin resistance (rpoB mutation), and streptomycin resistance (rpsL mutation) (Schrag et al., 1997; Maisnier-Patin et al., 2002; Silva et al., 2011; Wong, 2017). Specifically, mutations conferring resistance to antibiotics become advantageous when coexisting with other drug-resistant mutations or an additional resistance plasmid. Recognizing the power of sign epistasis, these antagonistic interactions highlight the necessity for more effective resistance reversal policies.

Lastly, insights into intra-genic sign epistasis also contribute to advanced protein design strategies, enhancing our understanding of protein evolution and conformation (Domingo et al., 2019; Starr et al., 2022).

## Discussion

In this review, we have delved into sign epistasis from a quantitative genetics perspective, offering a mechanistic understanding of the seemingly intricate interplay of effect-switching genetic mutations and their impacts across diverse biological systems. Understanding the mechanisms giving rise to sign epistasis, rooted in signalling cascades, peaked fitness landscapes, and physical Interactions (Table 1), contributes to advancing genotype-phenotype predictions and developing strategies to address challenges in agriculture (Blanc et al., 2006), antibiotic resistance (Wong, 2017), protein design (Miton and Tokuriki, 2016), and design of sophisticated genetic circuits (Baier et al., 2023).

**TABLE 1:** Summary of the mechanisms of sign epistasis.

Mechanism	Main feature	Type	Organism	Example	References
Hierarchy of signaling cascade	Without physical interactions, the combination of mutations from genes in upstream-dwonstream relationship shows sign-epistatic effects to mediate signaling cascade	Between genes	*E. coli*	In a linear hyrachial signalling cascade (arabinose senor, TetR and LacI regulators), the presence of TetR mutants alter sign of downstream Lac1 mutations	Nghe et al. (2018)
			*E. coli*	Within a three-nodes gene regulatory networks with arabinose sensor fused to TetR, a regulator LacI, and a reporter gene, different genotype combinations lead to sign epistatic effects and eventually diverse output	Baier et al. (2023)
Driven force of peaked fitness landscape	The nature of non-monotonic fitness landscape leads to non-additive phenotype combinations, which often lead to sign-changes of mutations	Between genes	*Methylobacterium extorquens*	Benificial mutations that alter enzyme levels interact antagonistically with each other to reach a balance between enzyme catalysis benefits and fitness costs	Chou et al. (2014)
			*E. coli*	Pairs of individually beneficial or deleterious mutations from araA and araB genes contribute to balance bewteen arabinose utilization and toxic accumulation of intermediates	Kemble et al. (2020)
		Within single gene	*E. coli*	Theorised that mutations in bacterial Lambda phage CI gene generate sign epistasis due to the peaked dose-response curve from pRM promoter	Li and Lehner, (2020)
Direct molecular contact	Often observed in compensotary mutations, where a second mutation restores the function of a molecule or a molecule complex by physically interacting with the first deterimental mutation	Between genes	*Bacillus amyloliquefaciens*	The E76R of barstar enables a salt bridge with barnase and partially compensates the interchange of the two charges caused by the R59E mutation in barnase	Jucovic and Hartley (1996)
			Bacteria	Direct interactions between bacterial toxin and antitoxin leads to preference of variants that serve as mutational intermediates	Aakre et al. (2015)
			*E. coli*	Sign epistasis of PhoQ interfacial residues alters PhoQ-PhoP binding and subsequent kinase activity	Podgornaia and Laub (2015)
			Human	Mutational effects in the alternatively spliced Fas exon 6 on mRNA splicing can switch their effect due to interaction between the pre-mRNA and splicing machinary	Baeza-Centurion et al. (2019)
			SARS-CoV-2	The reduction in ACE2 binding caused by Q498R mutation can be reversed by changing N501 by completing a salty bridge with ACE2	Starr et al. (2022)
		Within single gene	HIV-1	Detrimental L10I mutations enhances viral resistance to protease inhibitors in the presence of G48V and L90M	Mammano et al. (2000)

However, there is still a long way to go until we can leverage the knowledge of sign epistasis for the systematic engineering of biology. Besides the inherent challenges of predicting phenotypes posed by sign epistasis, the potential influence of the environment adds further complexity. It has been demonstrated that the occurrence or magnitude of sign epistasis can be altered by the environment, including the presence of inducers or inhibitors (Baier et al., 2023) and antibiotics (Ghenu et al., 2023). Nevertheless, the impact of other environmental factors on sign epistasis remains unclear.

In summary, unveiling the mechanisms of sign epistasis contributes to narrowing the gap between genotypes to phenotypes, providing insights into both the fundamental principles of molecular biology and practical applications across diverse fields.
